# From mechanisms to therapies: current advances breakthroughs in alopecia areata immunopathology

**DOI:** 10.3389/fimmu.2025.1621492

**Published:** 2025-09-01

**Authors:** Huai-Bo Zhao, Ya-Nan Zhang, Yan Qiang, Guo-Mi Wang, Li-Wei Wang, Wen-Cheng Jiang, Xi Chen

**Affiliations:** ^1^ Shanghai Skin Disease Hospital, School of Medicine, Tongji University, Shanghai, China; ^2^ Eight Departments of Traditional Chinese Medicine Surgery, Shanghai Municipal Hospital of Traditional Chinese Medicine, Shanghai University of Traditional Chinese Medicine, Shanghai, China

**Keywords:** alopecia areata, hair follicle immune privilege collapse, autoimmune, immunotherapy, targeted therapy

## Abstract

Alopecia areata (AA) is a prevalent autoimmune condition characterized by hair loss, with the collapse of hair follicle immune privilege being a pivotal event in its pathogenesis. This collapse involves intricate immunological disturbances, where CD8^+^NKG2D^+^ T cells, driven by inflammatory cytokines like IFN-γ, attack hair follicles. Additionally, various immune cell, including Th1, Th2, Th17 cells, γδT cells, NK cells, and mast cells, contribute to this pathological process. Defects in the function of Tregs, Bregs, and iNKT cells further compound the immune imbalance. At the molecular level, the JAK-STAT pathway emerges as a central regulatory node integrating multiple cytokine signals and presenting itself as a significant therapeutic target. JAK inhibitors have shown notable effectiveness in clinical settings, with some agents even gaining FDA approval for treating moderate-to-severe AA. However, the effectiveness of targeting IL-17, TNF-α, Th2 cytokines, PDE4, and other molecules remains debated. This review comprehensively explores the dynamic interactions among immune cell subsets, cytokine networks, and crucial signaling pathways in AA pathogenesis. It also summarizes the latest clinical progress and challenges in targeted therapies. Future studies should delve deeper into AA’s immune regulatory framework and devise tailored treatment approaches to enhance patient outcomes.

## 1 Introduction

Alopecia areata (AA) is a prevalent autoimmune-driven disorder marked by sudden-onset, non-scarring, and reversible hair loss. It often accompanies inflammatory or autoimmune comorbidities, such as atopic dermatitis, psoriasis, vitiligo, systemic lupus erythematosus, and Hashimoto’s thyroiditis (1, 2). Global AA incidence has escalated from 20.43 million cases in 1990 to 30.89 million cases in 2021, with notable prevalence in North America, Southeast Asia, and Australia, particularly affecting women and individuals aged 30–34 years (3). The clinical course of AA can exhibit spontaneous remission, recurrence, or exacerbation, significantly impacting patients’ quality of life and imposing substantial psychosocial burdens (4, 5).

A thorough understanding of the pathological mechanism of diseases can help explore more effective treatment methods (6). AA is characterized by a T cell-mediated autoimmune attack on hair follicles, leading to abrupt hair loss. Traditional therapies, including topical, intralesional, or systemic corticosteroids, have had variable success in suppressing this immune response (7–9), although patients with long-standing disease often experience high relapse rates (10). Contact immunotherapy agents like diphenylcyclopropenone offer safe and well-tolerated treatment options, albeit with limited efficacy (11, 12). Minoxidil may also be employed (13). Laser/phototherapy and superficial cryotherapy constitute effective and safe therapeutic avenues for AA and other diseases management (14–20). For severe AA patients who do not respond to other treatments or have contraindications to systemic corticosteroids, immunosuppressants such as methotrexate and cyclosporine may be considered. However, these treatments demonstrate suboptimal efficacy, elevated recurrence rates (21–23), and may cause side effects (24).

Recent research into AA’s pathogenesis has identified immune dysregulation as the central pathological mechanism. This involves the collapse of hair follicle immune privilege (HF-IP) and aberrant immune responses mediated by immune cells surrounding hair follicles, along with their produced cytokines and dysregulated Janus kinase-signal transducer and activator of transcription (JAK-STAT) signaling pathways. These advances have spurred the development of targeted therapeutic strategies, particularly JAK inhibitors and cytokine antagonists, showing promising clinical potential. This article comprehensively reviews recent progress in the immunological pathogenesis of AA, exploring emerging immunotherapeutic targets and related pharmacological agents. It aims to establish theoretical foundations and guide future research directions for AA treatment.

## 2 The immunological pathogenesis of alopecia areata

### 2.1 Collapse of hair follicle immune privilege

HF-IP denotes a defensive mechanism that shields hair follicles from immune detection (25). Under normal physiological conditions, hair follicles preserve their immune privilege through various regulatory mechanisms. The absence of lymphatic vessels, coupled with the perifollicular connective tissue sheath, forms physical barriers against immune cell infiltration (26, 27). The low expression of major histocompatibility complex (MHC)-I/II molecules helps evade T cell recognition (28, 29). CD200 communicates a ‘no danger’ signal to reduce antigen-presenting cell (APC) activity and suppress the secretion of pro-inflammatory cytokines (28, 30–32). The expression of programmed death ligand 1 (PDL1) in hair follicle cells contributes to immune privilege by directly modulating T cell function (29, 33). Low expression of major histocompatibility complex class I polypeptide-related sequence A (MICA) and UL16-binding protein (ULBP) hinders natural killer (NK) cell activation and natural killer cell group 2D (NKG2D) receptor-mediated recognition by CD8^+^ T cells (27). Additionally, the localized production of immunosuppressive factors, including α-melanocyte-stimulating hormone (α-MSH), transforming growth factor (TGF)-β1/2, interleukin (IL)-10, indoleamine 2,3-dioxygenase (IDO), macrophage migration inhibitory factor (MIF), calcitonin gene related peptide (CGRP), insulin-like growth factor-1 (IGF-1), thrombospondin 1 (TSP1), Fas ligand (FasL), and others, further bolsters follicular protection (29, 34, 35). Perifollicular mast cells, regulatory T cells (Tregs), and other immune cells may also play a role in maintaining HF-IP in healthy humans (28, 31, 36).

Genetic predisposition, viral infections or trauma, psychological stress, and other factors can trigger the breakdown of HF-IP (37, 38). The loss of this privilege is a prerequisite for the development of AA (39). Interferon (IFN)-γ-mediated upregulation of MHC class I and II antigen-presenting molecules is a key phenotypic change in HF-IP collapse, especially triggering CD8^+^ cytotoxic T lymphocytes cell-mediated autoimmune responses against follicular autoantigens associated with AA, such as tyrosinase-related protein, trichohyalin, melanoma antigen, retinol binding protein 4 (31, 40, 41). Enhanced MICA expression, accompanied by perifollicular infiltration of NKG2D^+^ NK cells and CD8^+^ T cells, decreased CD200 expression (28), reduced expression of immunosuppressive molecules (27), and multiple immune cells infiltration like mast cells stimulates immune responses in bodily tissues (36, 42), resulting in the loss of this protective mechanism. In the subsequent section, we will further explore the roles of immune cells and cytokines involved in HF-IP collapse in the development of AA.

### 2.2 Immune cells

#### 2.2.1 T cell subsets

##### 2.2.1.1 CD8^+^ T cells

The infiltration intensity of CD8^+^ T cells surrounding hair follicles in AA lesions demonstrates a notable correlation with disease severity (43). The transplantation of these cells has been found to induce localized alopecia in various animal models (28, 44). Furthermore, depleting CD8^+^ T cells in C3H/HeJ mice prevents and reverses AA, underscoring their essential role in the disease’s pathogenesis (45). CD8^+^ T cells target antigens presented by major histocompatibility complexes on hair follicles via T cell receptor (TCR), releasing IFN-γ and other pro-inflammatory cytokines that disrupt the HF-IP (27, 36, 46). Emerging evidence highlights CD8^+^NKG2D^+^ T cells as key regulators in AA pathogenesis. NKG2D, expressed on both CD8^+^ T cells and NK cells, functions as an activating receptor (30). Aberrant upregulation of NKG2D ligands, such as ULBP3/6 and MICA on keratinocytes facilitates the recruitment and activation of NKG2D^+^ CD8^+^ cytotoxic T cells (42). NKG2D interaction with its ligands can also enhance MHC expression (30). Studies have revealed that CD8^+^ T cells attack hair follicles via cytotoxic molecules, including granzyme B (GZMB) and perforin, produced by their cellular effector mechanisms (47, 48).

Additionally, virtual memory T (TVM) cells, a subset exhibiting a memory phenotype without prior foreign antigen exposure, have been implicated in AA. Recent research identifies CD44^super-high^CD49d^lo^CD8^+^ T cells as a novel subset derived from TVM cells. These cells can be induced through stimulation of conventional TVM cells with IL-12, IL-15, and IL-18. Their pathogenic activity relies on NKG2D receptor activation, which is further potentiated by IL-15 stimulation, ultimately leading to the onset of AA (49).

##### 2.2.1.2 CD4^+^ T cells

CD4^+^ T cells occupy a pivotal role in the development of AA, with imbalances and dysfunctional subsets collectively propelling disease advancement. A key histopathological hallmark of AA is the perifollicular infiltration of CD4^+^ T cells. Notably, elevated levels of NKG2D^+^ CD4^+^ T cells have been observed in the peripheral blood of AA patients (50). Animal models further underscore their pathogenic significance. In the C3H mouse model, the temporary depletion of CD4^+^ T cells triggers partial hair regrowth (29), whereas the adoptive transfer of CD4^+^CD25^-^ T cells from AA mouse skin draining lymph nodes (SDLNs) to healthy mice induces systemic alopecia (29, 51), highlighting the critical role of CD4^+^ T cell helper functions in AA pathogenesis (29).

In AA, T helper (Th)1, Th2, and Th17 cells all contribute to inflammatory reactions. Skin biopsy tissues reveal an augmented infiltration of C-C motif chemokine receptor (CCR) 5^+^ Th1 cells, while peripheral blood mononuclear cells (PBMCs) show elevated frequencies of CCR4^+^ Th2 cells (29). The increased population of skin-homing cutaneous lymphocyte-associated antigen (CLA)^+^ Th2 cells correlates with disease activity (37, 52), albeit the precise mechanisms remain elusive. CCR6^+^ Th17 cells not only circulate more but also densely infiltrate the hair bulb and peribulbar areas, exhibiting a more pronounced infiltration compared to CCR5^+^ Th1 cells (53, 54). Patients in active disease phases display significantly elevated Th17 levels, which inversely correlate with disease duration but bear no association with disease severity (55, 56). Th17 cells collaborate synergistically with Th1 cells to mediate inflammatory responses in AA. Moreover, as AA progresses, the balance between Th17 cells and Tregs becomes disrupted (57). AA patients in the active disease phase exhibit a higher proportion of Th17 cells relative to Treg cells in peripheral blood, indicating a predominance of pro-inflammatory responses. However, this trend reverses in severe AA patients, where Treg cell counts surpass those of Th17 cells, potentially reflecting the body’s compensatory mechanism to quell excessive immune responses in advanced disease stages (47).

Although Treg cells are crucial for maintaining hair follicle immune tolerance, AA patients exhibit reduced Treg populations accompanied by significant functional impairments. Specifically, AA patients showed notably lower percentages of forkhead box protein 3 (FoxP3)^+^CD4^+^ T lymphocytes in peripheral blood and decreased infiltration ratios of FoxP3^+^ lymphocytes in scalp tissues compared to healthy controls (29, 58). Paradoxically, the percentage of FoxP3^+^ cells in mild AA patients was even lower than in severe AA patients (29, 55). Selective depletion of Tregs in murine models exacerbates AA, affirming their protective role (45, 59). Nevertheless, merely expanding Tregs in AA mouse models fails to suppress CD8^+^ T cells and treat AA (60). Tregs primarily suppress Th1/Th2-mediated inflammatory responses via TGF-β and IL-10 secretion (61), and can directly regulate the activation of hair follicle stem cells (28). The functional defects of Tregs in AA patients may involve multiple mechanisms. Firstly, IL-6 and IL-1β in the inflammatory microenvironment hinder Treg differentiation and may drive Treg conversion to Th17 or cause a loss of their suppressive function (62). Secondly, AA patients display reduced expression of vital inhibitory molecules CD39 and human leukocyte antigen-DR (HLA-DR) on Treg surfaces (63). Additionally, genetic factors might contribute as Foxp3 gene polymorphisms could undermine Treg stability by decreasing mRNA expression (62).

In summary, the dysregulation of CD4^+^ T cell subsets, including the hyperactivation of Th1/Th2/Th17 and defects in both the quantity and function of Tregs, collectively undermines the HF-IP, thereby driving the pathogenesis and progression of AA.

##### 2.2.1.3 γδT cells

In skin affected by AA, γδT cells are present in both the bulbar and suprabulbar epithelia, with their numbers significantly elevated compared to healthy skin. When co-cultured with autologous stressed hair follicles overexpressing CD1d, C-X-C motif chemokine ligand (CXCL) 12, or MICA *in vitro*, γδT cells from healthy human scalp skin show induced expression of NKG2D and IFN-γ, leading to the development of AA-like pathological characteristics (64). Research has shown that most γδT cells encircling healthy hair follicles are non-activated γδ1^+^ T cells, bearing receptors such as C-X-C motif chemokine receptor (CXCR) 3, CXCR4, and CCR2. In AA-affected skin, however, these γδ1^+^ T cells adopt a pro-inflammatory phenotype, marked by elevated NKG2D and IFN-γ expression, coupled with reduced CD200 receptor (CD200R) levels. It’s noteworthy that IFN-γ-producing γδT cells are fewer in lesional skin than in non-lesional skin (65). In the C3H/HeJ mouse model of AA, depleting γδT cells did not halt or revert the progression of AA (45), suggesting that although γδT cells are involved in AA pathogenesis, they are not the major causative factor.

##### 2.2.1.4 Invariant natural killer T cells

The current consensus recognizes the protective role of invariant natural killer T (iNKT) cells in the pathogenesis of AA. Studies show that injecting iNKT cell activator, alpha-Galactosylceramide (α-GalCer) -stimulated natural killer T (NKT) cells or direct injection of α-GalCer into transplanted healthy human scalp skin in mice prevents the development of alopecia. However, when IL-10 is blocked in these skin grafts, NKT cells are unable to prevent alopecia, indicating that their protective effect on hair loss is dependent on IL-10. Furthermore, in AA, there is a notable numerical expansion of IL-10-producing iNKT cells, which have the capacity to suppress the proliferation and activity of NKG2D^+^CD8^+^ T cells (29, 66). This suppression may underlie the mechanism by which iNKT cells confer protection in AA.

#### 2.2.2 Natural killer cells

The precise role of NK cells in AA remains a topic of debate. Low MICA and NKG2D expression, coupled with high expression of killer immunoglobulin-like receptor (KIR) and MIF in hair follicles, effectively hinder the accumulation and assault of NK cells within and around these follicles (67). Lesional skin of AA patients shows an increased infiltration of CD56^+^NKG2D^+^ NK cells (50), which coincides with upregulated MICA and downregulated MIF expression in the follicles (68). Furthermore, patients experiencing complete alopecia have significantly higher proportions of CD57^-^CD16^+^ NK cells in their peripheral blood (69). These pathological shifts implicate NK cells in the autoimmune processes underlying AA. However, animal studies have surprisingly revealed that depleting NK cells can accelerate AA progression (29). Additional investigations have uncovered that this depletion results in a significant surge of CD49b^+^ T cell subsets within the lesional skin, subsets that exhibit pathogenic roles during early disease stages (70). These apparently conflicting observations suggest that NK cells may have subset-specific functions in AA. Specific NK cell subsets may exert protective effects on hair follicles via inhibitory signals such as KIR and MICA, while expressing NKG2D^+^ activated subsets are involved in autoimmune attacks. Overall, NK cells play a dual and complex role in AA, with their ultimate impact likely determined by a dynamic balance between surface receptor expression and microenvironmental factors.

#### 2.2.3 Dendritic cells

Multiple subsets of dendritic cells (DCs), including CD11c^+^ myeloid DCs, plasmacytoid DCs (pDCs), and CD1a^+^ Langerhans cells, are found around hair follicles in patients with AA (29, 40, 71). Subcutaneous injection of CD11c^+^ cells isolated from SDLNs of AA model mice into healthy C3H mice does not trigger disease onset in the recipients (51). Conversely, intradermal injection of pDCs effectively induces AA lesions (72). Activated pDCs producing IFN-α are detected in lesional skin and adjacent tissues of both AA patients and C3H/HeJ mouse models (72, 73). Studies on the underlying mechanisms reveal that activation of Toll-like receptors 7/9 (TLR7/9) on pDC surfaces stimulates potent IFN-α/β production, which then leads to the activation of CD4^+^ T cells, CD8^+^ T cells, and NK cells. This immune cascade initiates abnormal attacks on hair follicles, ultimately inhibiting hair growth (72). Furthermore, pDCs not only cause apoptosis of hair follicle epithelial cells but also boost the production of Th1/type 1 cytotoxic T cell (Tc1) chemokines such as CXCL10, attracting Th1/Tc1 cells and initiating autoimmune responses (72, 73). However, the precise mechanisms responsible for the recruitment of pDCs to hair follicles remain elusive (74, 75).

#### 2.2.4 Mast cells

The role of mast cells in the pathogenesis of AA has been increasingly clarified. Notably, significant mast cell infiltration occurs in lesional areas, both in patients with AA and in the C3H mouse model (29, 71). Furthermore, these cells undergo substantial functional changes, including a reduced immunosuppressive capacity due to decreased TGFβ1 expression (28) and heightened pro-inflammatory activity, as evidenced by elevated tryptase expression and degranulation (76). Crucially, activated mast cells engage in close interactions with autoreactive CD8^+^ T cells through the upregulation of co-stimulatory molecules like OX40 ligand (OX40L), CD30 ligand (CD30L), 4-1BB ligand (4-1BBL), and intercellular adhesion molecule 1 (ICAM1) (76), thereby contributing to the breakdown of immune privilege and facilitating antigen presentation mechanisms (42). However, research suggests no correlation between the course or activity of AA and peribulbar mast cell infiltration (77).

#### 2.2.5 Type 1 innate lymphoid cells

Type 1 innate lymphoid cells (ILC1) show a marked increase around lesional and non-lesional hair follicles in AA patients. Both *in vivo* and *in vitro* investigations reveal that IFN-γ-producing NKG2D^+^ ILC1 cells have the capacity to trigger distinct AA lesions and disrupt the HF-IP (78).

#### 2.2.6 Regulatory B cells

During the progression of AA, regulatory B cells (Bregs) that produce IL-10 might exert a protective function. Research indicates a notable decrease in the count of IL-10-producing Breg cells among PBMCs of AA patients. These Breg cells, specifically the CD19^+^CD24^hi^CD38^hi^ subtype, have the capacity to down-regulate NKG2D^+^CD8^+^ T cells and IFN-γ secretion, ultimately damping down exaggerated immune reactions. Nevertheless, certain investigations have detected elevated IL-10 levels in the B cells of AA patients relative to healthy controls. This apparent contradiction could stem from augmented compensatory negative feedback loops operating within the diseased state (79).

### 2.3 Cytokines and related signaling pathways

#### 2.3.1 Related cytokines and chemokines

The pathogenesis of AA involves a complex network of cytokines, with Th1-type immune responses and common gamma chain (γc) cytokines playing pivotal roles. As a key cytokine in Th1 responses, IFN-γ shows marked elevation in the serum of AA patients, correlating closely with disease activity and clinical course duration (30, 58). Produced not only by Th1 cells but also by ILC1, NK cells, NKT cells, and γδT cells (36), IFN-γ induces MHC class I molecule expression, MICA expression, and the production of CXCR3 ligands CXCL9/10/11 (28, 38, 48, 80, 81). This recruitment of CXCR3^+^ Th1 cells, CD8^+^ T cells, NK cells (61) is accompanied by stimulation of IL-2 and IL-15 production, further activating CD8^+^ T cells (82–84). These immune cells activation leads to a persistent production of additional IFN-γ, establishing a positive feedback loop (47, 81) that directly contributes to the collapse of HF-IP (29, 40). Tumor necrosis factor (TNF)-α expression is elevated in AA patients’ serum (85, 86) promoting inflammation through upregulation of MHCI protein expression in dermal papilla cells (87).

Within the γc cytokine family, IL-2, secreted by DCs, NK cells, CD4^+^, and CD8^+^ T cells (28), promotes the infiltration of CD4^+^, and CD8^+^ T cells into hair follicles (88). IL-2 also participates in Treg cell homeostasis, with low-dose IL-2 promoting Treg cell proliferation and restoring the Th17/Treg cell balance. However, the limited efficacy of low-dose IL-2 injection therapy in severe AA suggests its primary pro-inflammatory role in this context (89). Inhibition of both IFN-γ and IL-2 can halt disease progression (47, 68, 90). Skin biopsy tissue from AA patients exhibits significantly higher numbers of IL-15^+^ T cells around hair follicles compared to healthy controls (29), while IL-15, interleukin-15 receptor (IL-15R) α, and IL-15Rγ protein expression within the hair bulbs of AA lesions is downregulated (91). IL-15 exacerbates tissue damage by activating NKG2D^+^CD8^+^ T cells and NKG2D^+^ NK cells, while simultaneously impairing Treg functionality (28, 29). Inhibition of IL-15Rβ can prevent disease progression (47, 68).Recent studies reveal that recombinant human IL-15 significantly downregulates MICA expression in the hair bulb, promotes α-MSH production, protect iNKT10 cells from IFN-γ-induced apoptosis, and facilitates hair regeneration through IL-15Rα-dependent signaling (91). Selective activation of the local IL-15Rα signaling pathway in hair follicles may become a new strategy for the treatment of AA. Other γc cytokines such as IL-7 may also be involved in the pathogenesis of AA (92).

Th2-related cytokines, including IL-4, IL-5, IL-13, and associated chemokines such as C-C motif chemokine ligand (CCL) 13, CCL17, and CCL26, are elevated in AA patients (29, 37, 40, 93, 94). Additionally, the expression of type 2-related biomarkers CCL18, thymic stromal lymphopoietin (TSLP), and IL-9 was increased in the skin lesions, and serum IL-6, immunoglobulin E, and eosinophils were elevated (38, 95, 96). CCL17 can serve as a biomarker for disease activity and treatment response (97). The roles of these cytokines and chemokines in AA are still being investigated. Elevated levels of Th17-related cytokines IL-17 and IL-22 have been observed in serum and tissues of AA patients (38, 57, 98–100), and the Th17 pathway may interact synergistically with the Th1 pathway to promote disease progression (61, 101). IL-12 and IL-23 are cytokines produced by DC, which respectively induce the differentiation of Th1 and Th17. Although the expression of the common subunit p40 of IL-12/23 in AA lesions was increased compared with that in normal skin and non-lesions (29). However, studies have shown that neutralization of the shared IL-12/23 p40 subunit with specific antibodies failed to prevent AA development in the C3H/HeJ mouse model (29, 54), and IL-12/23 inhibitors for psoriasis even induce AA (102, 103), suggesting that the classical Th17 differentiation pathway mediated by IL-12/23 may not be the primary driver in AA pathogenesis.

Other factors, such as elevated expression of tumor necrosis factor superfamily members like tumor necrosis factor-related weak inducer of apoptosis (TWEAK) (104) and a possible association between interleukin-1 receptor type 1 (IL-1R1) and AA development and disease activity (105), indicate potential involvement of additional inflammatory pathways. Recent studies also suggest CXCL12 as a potential therapeutic target for AA, as humanized CXCL12 neutralizing antibody delayed disease onset through reducing the infiltration of T cells, DC and macrophages, down-regulating IFN-γ pathway-related genes such as IFN-γ, CD8a, and CCR5, and inhibiting the abnormal activation of the JAK/STAT pathway and CXCR4 signaling pathway in murine AA models (106).

Inhibitory cytokines such as IL-10, TGF-β, and IL-35, primarily secreted by Breg and Treg cells, maintain immune tolerance homeostasis through multiple immunosuppressive pathways. IL-10 can down-regulate the expression of MHC Class II molecules on the surface of APCs and weaken their antigen-presenting ability (62). TGF-β can not only down-regulate the expression of MHC-I, inhibit T cell activation and APCs activity, but also maintain the expression of Foxp3 in Treg cells (28, 62). IL-35 regulates the immune response by restricting T cell proliferation (62). The synergistic action of these cytokines constitutes a crucial molecular basis for suppressing autoimmune attacks.

In conclusion, the immune dysregulation observed in AA exhibits mixed characteristics, predominantly featuring IFN-γ-driven Th1 polarization. Further investigation is needed to elucidate the roles of other cytokines and chemokines in AA pathogenesis, and targeted interventions addressing key cytokines may offer promising therapeutic avenues.

#### 2.3.2 JAK-STAT signaling pathway

The JAK-STAT pathway occupies a central position in the pathogenesis of AA, emerging as a potential therapeutic target (107). Pro-inflammatory cytokines linked to AA, such as IFN-γ, IL-2, IL-15, IL-7, and IL-21, interact with their cognate receptors, triggering the JAK-STAT signaling cascade and disrupting the hair follicles’ growth cycle (46, 108, 109). The JAK family, consisting of JAK1, JAK2, JAK3, and tyrosine kinase 2 (TYK2), operates as cytoplasmic tyrosine kinases critical for signal transduction in both type 1 and type 2 cytokine receptors (110). Upon cytokine binding, receptor dimerization occurs, leading to JAK protein transphosphorylation, followed by STAT protein (including STAT1, STAT2, STAT3, STAT4, STAT5a, STAT5b, and STAT6) phosphorylation and dimerization. These activated STAT proteins then migrate to the nucleus, binding to specific DNA sequences and regulating the expression of downstream genes, thus influencing the immune environment of hair follicles. The downstream effects of cytokines signaling through the JAK/STAT pathway are determined by both the specific ligand activating the pathway and the unique combinations of JAK kinase subtypes associated with the receptors. Distinct cytokine-receptor complexes recruit particular JAK kinase combinations, activating specific STAT proteins that drive unique gene expression patterns and biological outcomes. For instance, when IFN-γ binds to its receptors on hair follicle epithelial cells, it stimulates IL-15 production via the JAK1/2-STAT pathway. IL-15 subsequently activates CD8^+^ T cells through the JAK1/3-STAT pathway, promoting further IFN-γ release and creating a positive feedback loop that intensifies inflammatory damage to hair follicles (83, 111). IL-7 exerts its effects through JAK1/JAK3 (92). Cytokines such as IFN-α, IFN-γ, IL12/23, IL-6, and IL-10 mediate regulatory roles via TYK2 (111). JAK3 specifically partners with common γ-chain receptors (111). Clinical studies have shown that inhibiting the JAK/STAT signaling pathway can alleviate alopecia symptoms and foster hair regrowth (112).

#### 2.3.3 Phosphodiesterase 4-mediated immunoregulatory functions

Study have discovered that phosphodiesterase 4B is among the differentially expressed genes found in both lesional and non-lesional areas of AA patients, as well as in healthy controls. This finding provides initial evidence suggesting phosphodiesterase 4 (PDE4)’s potential involvement in the development of AA. PDE4 enzymes are known to break down cyclic adenosine monophosphate (cAMP), a signaling molecule regulating inflammatory reactions within cells. By inhibiting PDE4, intracellular cAMP concentrations rise, leading to a reduction in the production of inflammatory cytokines such as TNF-α, IL-23, and IFN-γ, which is crucial for maintaining healthy hair follicles. Although PDE4 has emerged as a potential therapeutic target for new drug development in AA, current clinical trials have not shown promising results, indicating that the precise role of PDE4 in the pathogenesis of alopecia areata remains uncertain (95).

#### 2.3.4 TEC family kinases-mediated immunoregulatory functions

The TEC kinase family belongs to the class of non-receptor tyrosine kinases and includes five members: TEC, Bruton’s tyrosine kinase (BTK), IL-2-inducible T-cell kinase (ITK), resting lymphocyte kinase (RLK, also known as TXK), and bone marrow tyrosine kinase on chromosome X (BMX) (113). The role of ITK in AA has been extensively studied. Upon TCR recognition of antigens and subsequent activation, ITK kinase joins the signaling complex, undergoing phosphorylation and activation. Once activated, ITK phosphorylates phospholipase Cγ1 (PLCγ1), enabling it to gain catalytic activity. PLCγ1 then hydrolyzes phosphatidylinositol 4,5-bisphosphate (PIP2) on the cell membrane, producing two essential second messenger molecules: inositol trisphosphate (IP3) and diacylglycerol (DAG). Together, IP3 and DAG stimulate calcium influx and aid in the nuclear translocation of transcription factors, enhancing the transcriptional expression of inflammatory cytokines like IFN-γ and IL-17, ultimately triggering the immune response (114).

#### 2.3.5 Aire-mediated immunoregulatory functions

Aire, a transcriptional regulatory protein, plays a pivotal role in maintaining immune tolerance. Research indicates that mice lacking Aire (Aire^-/-^) spontaneously exhibit persistent AA-like lesions, which are accompanied by a disruption of HP-IP. Notably, significant infiltration of CD8^+^ T cells, CD4^+^ T cells, CD68^+^ macrophages, and mast cells is observed in the vicinity of these lesional hair follicles. On a molecular level, Aire deficiency results in the upregulation of MHC molecules, downregulation of α-MSH, and induces excessive expression of IFN-γ and its downstream chemokines, such as CCL5 and CXCL9/10/11. Additionally, this deficiency triggers the hyperactivation of JAK-STAT signaling (115).

#### 2.3.6 Other signaling

The nuclear factor-kappa B (NF-κB) pathway and NOD-like receptor family pyrin domain containing 3 (NLRP3) inflammasome activation are involved in the pathogenesis of AA. MCC950, an NLRP3 inhibitor, has been found to hinder AA development in murine models and stimulate hair regrowth (116, 117). Additionally, the serine/threonine kinase, PTEN induced kinase 1 (PINK1)-mediated mitophagy, mitigates inflammatory responses by suppressing NLRP3 inflammasome activation (118). Additionally, receptor-interacting protein kinase 1 (RIPK1) plays a part in AA pathogenesis through immune cell regulation, with increased expression observed in DCs and CD8^+^ T cells in AA mouse models. Inhibitors of RIPK1 can postpone AA onset and decrease the infiltration of these cells in the skin (119). Furthermore, the zinc finger transcription factor Ikaros demonstrates elevated expression in AA patients, and transgenic mouse experiments have shown that Ikaros overexpression induces phenotypes similar to AA, implying its role in the disease’s pathogenesis (120, 121). Sirtuin 1 (SIRT1) expression is notably decreased in AA-affected scalp tissue. Inhibition of SIRT1 suppresses MICA and ULBP3, while promoting the production of inflammatory cytokines, such as IFN-γ, TNF-α, CXCL9, CXCL10, and enhancing T cell migration. Conversely, activating SIRT1 suppresses autoreactive inflammatory responses (122). Other signaling pathways, including wingless-integrated/beta-catenin (Wnt/β-catenin), mitogen-activated protein kinase (MAPK), and Ras, may also be implicated in the pathogenesis of AA (123–125).

**Figure 1:**
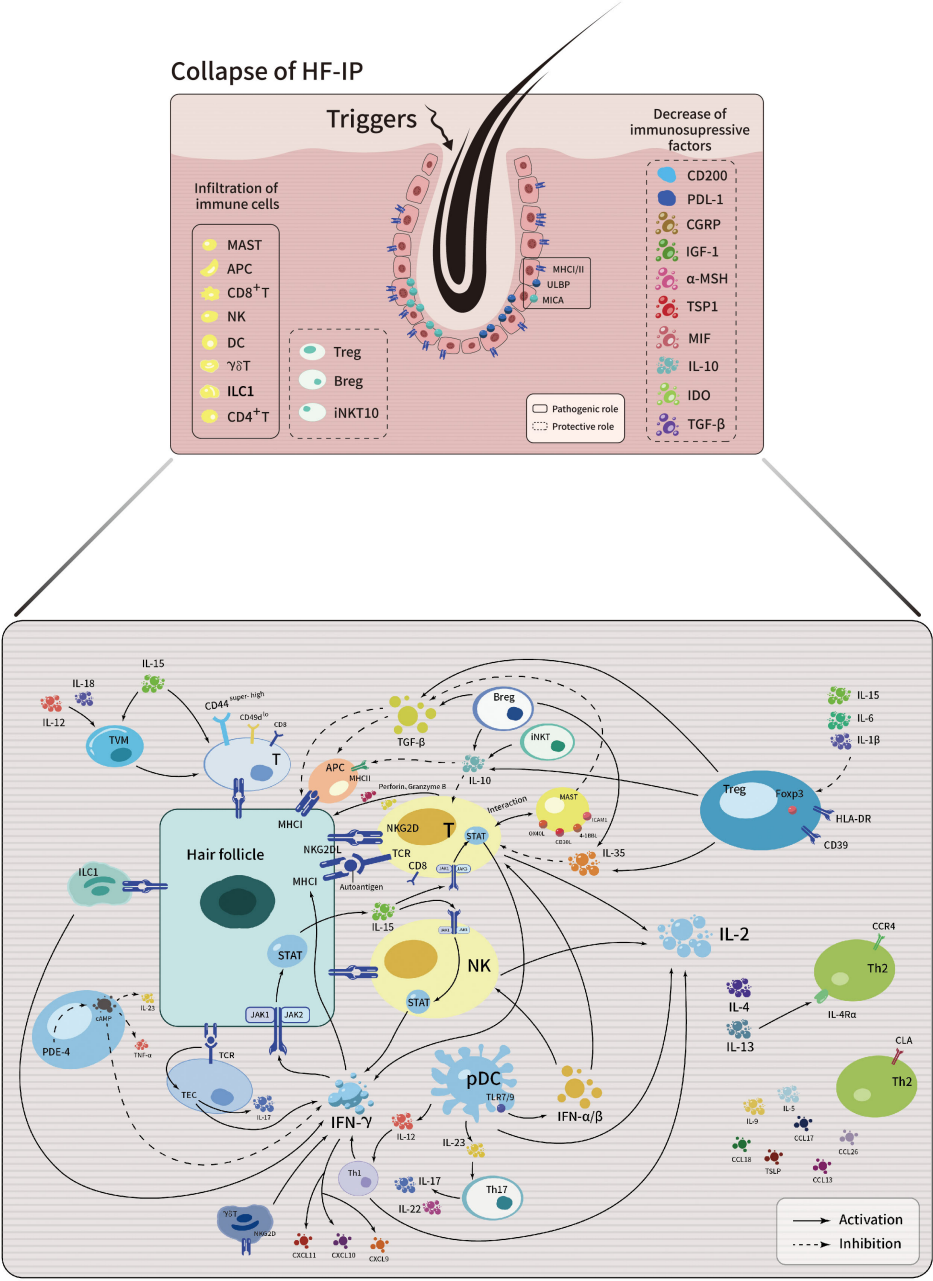
Schematic illustration depicting the intricate interplay among immune cells, cytokines, chemokines and signaling pathways in the development of AA.

The summary of AA pathogenesis discussed in this article is schematically illustrated in **Figure 1**.

## 3 Novel strategies in immune-targeted therapy

### 3.1 JAK inhibitors

JAK inhibitors, small molecules targeting Janus kinases to disrupt signaling, have become a pivotal research focus in AA therapy. The U.S. Food and Drug Administration (FDA) has approved three such inhibitors: baricitinib, deuruxolitinib (CTP-543), and ritlecitinib, for the treatment of this condition (111, 126, 127). Baricitinib and deuruxolitinib, primarily JAK1/2 inhibitors, are indicated for severe AA in adults. Baricitinib also exhibits JAK3 inhibitory activity and has proven long-term efficacy and safety in several studies (128, 129). Its therapeutic effect surpasses conventional treatments (130). Baricitinib also shows promise in patients aged 65 and above (131). A phase 3 double-blind RCT (NCT05723198) evaluating baricitinib in children aged 6 to 18 years with AA is actively recruiting participants. Besides, A Phase 2 prospective open-label study (NCT06797310) to evaluate the effectiveness and tolerance of baricitinib in acute AA with active hair shedding is planned. Deuruxolitinib effectively reduces AA severity (132). Phase 3 randomized controlled trials (RCTs) revealed that 41.5% of adult severe patients receiving deuruxolitinib achieved a Severity of Alopecia Tool (SALT) score of 20 or less (133). Ritlecitinib, a selective dual inhibitor targeting JAK3 and TEC kinases, is indicated for severe AA in patients aged 12 and older (134–137). Its safety and efficacy have been confirmed in numerous trials (135, 138–140). Recently, a phase 3 randomized study (NCT06873945) with a dose-up strategy for non-responders to evaluate the safety and efficacy of ritlecitinib 50 mg and 100 mg once daily in participants aged ≥12 years with severe AA is underway. Two phase 3 clinical trials (NCT07029711, NCT07029828) will soon assess ritlecitinib for the treatment of severe AA in children aged between 6–12 and 6–14 years, respectively.

Tofacitinib and ATI-501, JAK1/3 inhibitors, demonstrate effectiveness and tolerability in AA treatment, as evidenced by multiple cohort studies and RCTs (141–147). Tofacitinib achieved a 33.8% complete response rate after 24 weeks (148). Among pediatric patients unresponsive to previous therapies, an 87% efficacy rate was observed (149). ATI-501 showed a 30.4% improvement in SALT score at 24 weeks in a phase 2 RCT (150). Ruxolitinib, JAK1/2 inhibitors, also show promise. In an open-label trial, ruxolitinib achieved a 92% mean hair regrowth rate (151). Additionally, KL130008, a novel selective inhibitor of JAK 1/2, is under investigation in a phase 2 multicenter, double-blind, RCT (NCT05496426), though its current status remains unknown. Brepocitinib, a TYK2/JAK1 inhibitor, significantly improved SALT and Alopecia Areata Symptom Impact Scale (AASIS) scores compared to placebo in RCTs (136, 152, 153). Deucravacitinib, a selective TYK2 inhibitor, showed hair regrowth in two severe AA case reports (154, 155). While, a phase 2 study (NCT05556265) to evaluate its efficacy and safety in AA participants was terminated due to change in business objectives. Ivarmacitinib (SHR0302), a selective JAK1 inhibitor, demonstrated higher responder rates of absolute SALT20 (47.8%) than placebo (20.8%) in treating moderate-to-severe AA at week 24 in a phase 2 double-blind RCT (156). The confirmatory phase 3 trial (NCT05470413) is currently in the Not Yet Recruiting status. Case reports suggest that highly selective JAK1 inhibitors, including upadacitinib, abrocitinib, and filgotinib, may also be beneficial (157–163). Two phase 3 double-blind, RCTs (NCT07023302, NCT06012240) is currently underway to evaluate the safety and efficacy of upadacitinib oral tablets in adult and adolescent participants with AA. A single-center, real-world study (NCT06573593) is investigating the efficacy and safety of JAK inhibitors including abrocitinib in treating AA patients. Additionally, a phase 2 RCT (NCT06562894) focusing on the use of SYHX1901, JAK 1/2/3 and TYK2 inhibitors, for the treatment of severe AA is anticipated to commence soon.

Topical JAK inhibitors show limited efficacy (111, 150, 164). However, combining topical JAK inhibitors like tofacitinib with minoxidil demonstrates synergistic potential by modulating the IFN-γ/IL-4 ratio (165).

For patients with severe AA alone, JAK inhibitors should be prioritized as first treatment strategy over other immune-targeted agents, particularly utilizing FDA-approved agents such as baricitinib, ritlecitinib, or deuruxolitinib.

### 3.2 Th2 cytokine inhibitors

Dupilumab, a monoclonal antibody that targets IL-4Rα, exerts its therapeutic effects by blocking both IL-4 and IL-13 signaling pathways. While current clinical studies on dupilumab’s efficacy in treating AA have yielded mixed results, there have been reports of dupilumab-induced AA cases (166, 167). Nevertheless, a few studies and case reports have also shown dupilumab to be effective in treating AA. In a phase 2 randomized controlled trial, after 48 weeks of treatment with dupilumab, 32.5%, 22.5%, and 15% of patients met the improvement criteria for SALT30, SALT50, and SALT75, respectively (168). A phase 2 double-blind RCT (NCT05866562) evaluating dupilumab in pediatric AA patients aged 6–17 years, is currently recruiting. Notably, more pronounced therapeutic effects were observed in patients with atopic AA (169, 170). However, for AA patients without atopic comorbidities, dupilumab’s therapeutic efficacy was limited. This limitation may be due to its downregulation of the Th2 immune response, which relatively enhances Th1 immune reactivity, potentially triggering or worsening AA (166). Similarly, while a case report indicated that the anti-IL-13 monoclonal antibody tralokinumab demonstrated efficacy in resolving both atopic dermatitis and AA in a patient with concurrent disease (171), a phase 2 randomized placebo-controlled pilot study (NCT02684097) of tralokinumab in moderate-to-severe AA showed high treatment discontinuation (13/15 patients) due to lack of efficacy, with neither of the two completing patients achieving SALT50. These findings suggest that the therapeutic efficacy of Th2-targeted biologics in AA may be influenced by the patient’s baseline immune status, specifically the balance between Th1 and Th2 immune responses. Consequently, Th2-targeted therapies should primarily be considered for AA patients with coexisting atopic diseases, whereas alternative approaches may be more appropriate for isolated AA cases. Future clinical trials focusing on patients with pure AA (without concurrent atopic diseases) are needed to elucidate the role of the Th2 immune response in AA pathogenesis and to more precisely define the therapeutic value of these biologics across different AA subtypes.

### 3.3 IL-12/IL-23 inhibitors

Although the specific role of IL-12/IL-23 in the development of AA is not fully understood, several case reports have highlighted the potential of IL-12/IL-23 inhibitors as a therapeutic approach. In particular, ustekinumab, which targets the IL-12/IL-23 p40 subunit, has been reported to induce hair regrowth in all of the six patients with AA (172, 173). These findings suggest that ustekinumab could be a viable treatment option for those who have not responded to conventional therapies. Additionally, tildrakizumab, a specific IL-23 p19 inhibitor, has been investigated in a pilot study for the treatment of moderate-to-severe AA. However, the study observed partial hair regrowth in only two out of nine patients (174), indicating that its efficacy may be more limited. Given the limited clinical evidence for IL-12/IL-23 inhibitors, these agents require cautious use in pure AA cases. Ustekinumab represents a potential option for AA patients unresponsive to conventional therapies or those with concurrent moderate-to-severe plaque psoriasis, active psoriatic arthritis, or moderate-to-severe active Crohn’s or ulcerative colitis. Further research is needed to determine the most effective agents and treatment strategies for different subpopulations.

### 3.4 IL-17 inhibitors

IL-17 inhibitors, which are monoclonal antibodies targeting IL-17A or its receptor, have shown remarkable effectiveness in the treatment of psoriasis. Nevertheless, recent research reveals that certain psoriasis patients developed AA symptoms between 2 to 13 months after commencing treatment with IL-17 inhibitors like brodalumab, secukinumab, and ixekizumab (175). This observation suggests a possible link to the drugs’ mode of action: by potentially disturbing the delicate Th1/Th17 immune balance, these inhibitors might trigger an abnormal upregulation of the Th1 pathway. This, in turn, could induce perifollicular inflammatory reactions, thus facilitating the development of AA (175). Disappointingly, despite attempts to clinically apply anti-IL-17 agents in the treatment of AA, significant therapeutic benefits have yet to be demonstrated (176). The use of these medications is avoided in patients with pure AA or AA comorbid with conditions for which these drugs are already indicated, such as psoriasis, psoriatic arthritis, or ankylosing spondylitis.

### 3.5 TNF-α inhibitors

Etanercept, a TNF-α inhibitor, has demonstrated restricted therapeutic effectiveness in the treatment of AA during clinical trials. A prospective investigation assessing 17 individuals with moderate-to-severe AA who received etanercept therapy revealed that none of them attained SALT score improvements surpassing 10%. In fact, certain patients observed disease progression (177). Etanercept should not be used to treat AA alone, but it may be appropriate when AA occurs alongside rheumatoid arthritis, ankylosing spondylitis, psoriatic arthritis or plaque psoriasis, where TNF-α plays a central role in disease pathogenesis.

### 3.6 PDE4 inhibitors

Studies have demonstrated that apremilast, a PDE4 inhibitor, hinders the development of AA in humanized mouse models (178). Clinical trial findings, however, have been not consistent. While several reports suggest that apremilast has limited effectiveness in treating AA (179–181), a retrospective analysis revealed notable results. Specifically, out of 15 patients with refractory AA who did not respond to standard treatments, 13 experienced over 50% hair regeneration after receiving apremilast (182). Additional case reports also bolster its therapeutic potential (183). Nevertheless, more clinical trials are necessary to confirm its efficacy. Furthermore, a completed randomized early Phase 1 clinical trial (NCT06527729) evaluated a novel sildenafil (PDE5 inhibitors)-loaded nanocarrier formulation for AA, though study results remain unpublished. For patients with refractory AA unresponsive to conventional therapies like JAK inhibitors, or those presenting with comorbid plaque psoriasis or psoriatic arthritis, apremilast represents a viable alternative.

### 3.7 Other strategies in immune-targeted therapy

Two randomized, double-blind, placebo-controlled clinical trials (including study NCT00167102) investigating alefacept, a T-cell biologic inhibitor, for the treatment of severe AA found it ineffective (184). In a phase 2 open-label, single-arm clinical trial (NCT02018042) of abatacept, a CD80/CD86 inhibitor that blocks T cell co-stimulation, administered to 15 patients with moderate-to-severe patchy AA, alopecia totalis, or alopecia universalis, one patient achieved the primary endpoint of >50% hair regrowth. The phase 2 double-blind RCT (NCT05205070) evaluating rosnilimab (ANB030), PD-1 agonists, in moderate-to-severe AA currently has unknown study status. Etrasimod, an oral selective sphingosine 1-phosphate (S1P) receptor modulator approved for ulcerative colitis, failed to meet primary and secondary efficacy endpoints in a Phase 2 multicenter, double-blind RCT (NCT04556734) in adults with moderate-to-severe AA, where the mechanistic role of S1P signaling remains poorly characterized. Another S1P receptor modulator NXC-736, is currently undergoing a phase 2a RCT (NCT06104839). In the phase 2a open-label proof-of-concept trial (NCT05368103) evaluating daxdilimab, an anti-IL-2 receptor β chain (CD122) monoclonal antibody, for moderate- to-severe AA, 20% of patients achieved SALT50 at 24 weeks.

Some novel agents are under investigation in AA clinical trials: HCW9302 (IL-2 fusion protein, NCT07049328), rezpegaldesleukin (Treg-selective IL-2 receptor agonist, NCT06340360), IMG-007 (OX40 inhibitor, NCT06060977), amlitelimab (OX40L inhibitor, NCT06444451), farudodstat (pyrimidine biosynthesis inhibitor, NCT05865041), and bempikibart (IL-7Rα inhibitor, NCT06018428), DR-01 (NCT06602232), VIS171 (NCT06799520) and ALD-102 solution (NCT06826196).

Novel immune-targeted therapeutic strategies for AA are summarized in **Table 1**.

**Table 1:** Novel strategies in immune-targeted therapy.

Targeted therapy	Management options	Targets	Current clinical trials
JAK inhibitors	Baricitinib	JAK1/2/3	FDA-approved for severe AA in adults Phase 3 double-blind RCT (NCT05723198): for children aged 6 - 18 years with AA (ongoing) Phase 2 open-label (NCT06797310): for acute AA with active hair shedding (ongoing)
	Deuruxolitinib	JAK1/2	FDA-approved for severe AA in adults
	Ritlecitinib	JAK3, TEC	FDA-approved for severe AA in patients aged ≥12 years Phase 3 RCT (NCT06873945): dose-up in severe AA ≥12 years (ongoing) Phase 3 Trials in children with severe AA: 6–12 years (NCT07029711); 6–14 years (NCT07029828) (ongoing)
	Tofacitinib	JAK1/3	Shows effectiveness in RCT, multiple cohort studies and case reports (141–149)
	ATI-501	JAK1/3	Shows a 30.4% improvement in SALT score in a phase 2 RCT (150)
	Ruxolitinib	JAK1/2	Shows high mean hair regrowth rate in an open-label trial (151)
	Brepocitinib	TYK2, JAK1	Shows effectiveness in RCTs (136, 152, 153)
	Deucravacitinib	TYK2	Shows hair regrowth in two severe AA case reports (154, 155) Phase 2 study (NCT05556265): for AA participants (terminated due to change in business objectives)
	Ivarmacitinib	JAK1	Shows beneficial impact in a phase 2 RCT (156) Phase 3 RCT (NCT05470413): for adults with severe AA (ongoing)
	Upadacitinib	JAK1	Shows effectiveness in a cohort study and case reports (157–160) Phase 3 RCTs (NCT07023302, NCT06012240): oral tabs for AA in adults/adolescents (ongoing)
	Abrocitinib	JAK1	Shows effectiveness in two case reports (161, 162) Real-world study (NCT06573593): for patients with AA (ongoing)
	Filgotinib	JAK1	Shows effectiveness in a case report (163)
	Delgocitinib	JAK1/2/3, TYK2	A small-scale RCT demonstrates no significant efficacy of topical therapy in moderate-to-severe patients (164)
	KL130008	JAK1/2	Phase 2 double-blind, RCT (NCT05496426): for adults with severe AA (unknown status)
	SYHX1901	JAK1/2/3, TYK2	Phase 2 double-blind RCT (NCT06562894): for severe AA (ongoing)
Th2 cytokine inhibitors	Dupilumab	IL-4Rα	With conflicting evidence (166–170) Phase 2 double-blind RCT (NCT05866562): for pediatric AA patients aged 6–17 years (ongoing)
	Tralokinumab	IL-13	Phase 2 randomized placebo-controlled pilot study (NCT02684097) demonstrates lack of efficacy in moderate-to-severe AA
IL-12/IL-23 inhibitors	Ustekinumab	IL-12/IL-23 p40 subunit	Induce hair regrowth in case reports (172, 173)
	Tildrakizumab	IL-23 p19 subunit	Partial hair regrowth in two out of nine patients (174)
IL-17 inhibitors	Brodalumab	IL-17RA	AA develops after treatment in psoriasis patients (175)
	Secukinumab	IL-17A	Demonstrates no significant benefits in a double-blinded, randomized prospective pilot study (176)
	Ixekizumab	IL-17A	AA develops after treatment in psoriasis patients (175)
TNF-α inhibitors	Etanercept	TNF-α	Shows restricted therapeutic effectiveness in a small prospective investigation (177)
PDE4 inhibitors	Apremilast	PDE4	With conflicting evidence (179–183)
PDE5 inhibitors	Sildenafil	PDE5	Early Phase 1 randomized trial (NCT06527729): Sildenafil -loaded nanocarrier formulation for AA (completed)
Other strategies	Alefacept	CD2 T-cell receptor	Shows ineffectiveness in 2 RCTs (NCT00167102) (184)
	Abatacept	CD80/CD86	Phase 2 open-label trial (NCT02018042) reported that 1 patient achieved >50% hair regrowth among 15 patients with moderate-to-severe AA
	Rosnilimab	PD-1 agonist	Phase 2 RCT (NCT05205070): for moderate-to-severe AA (unknown status)
	Etrasimod	S1P	Primary and secondary efficacy endpoints are not met in a phase 2 RCT (NCT04556734) in adults with moderate-to-severe AA
	NXC-736	S1P	Phase 2a RCT (NCT06104839): for adult participants with moderate-to-severe AA (ongoing)
	Daxdilimab	IL-2 receptor β chain (CD122)	20% of patients with moderate-to-severe AA achieved SALT50 in the phase 2a open-label proof-of-concept trial (NCT05368103)
	HCW9302 (IL-2 fusion protein)	IL-2	Phase 1, open-label, and dose-escalation study (NCT07049328): for AA subjects (ongoing)
	Rezpegaldesleukin	Treg-selective IL-2 receptor agonist	Phase 2b, double-blind, parallel group, RCT (NCT06340360): for severe to very severe AA adults (ongoing)
	IMG-007	OX40	Phase 1b/2a open label study (NCT06060977): for adults with severe AA (completed)
	Amlitelimab	OX40L	Phase 2 double-blind, parallel group, 3-arm, proof-of-concept RCT (NCT06444451): for adult with severe AA (ongoing)
	Farudodstat	Pyrimidine biosynthesis	Phase 2a, double-blind, two-arm study (NCT05865041): for adult AA (ongoing)
	Bempikibart	IL-7Rα	Phase 2a RCT (NCT06018428): for the treatment of severe AA (ongoing)
	DR-01	/	Phase 1b pilot study (NCT06602232): for subjects with AA (ongoing)
	VIS171	/	Phase 1 open-label trial (NCT06799520): for AA participants (ongoing)
	ALD-102 solution	/	Phase 1b/2a double-blind, intraindividual RCT (NCT06826196): for AA subjects (ongoing)

## 4 Conclusions

Alopecia areata, an autoimmune disease characterized by hair loss, is rooted in the breakdown of the HP-IP. Its pathogenesis involves a complex dysregulation of the immune system, wherein CD8^+^NKG2D^+^ T cells, driven by pro-inflammatory cytokines like IFN-γ, attack the hair follicles. This immune dysregulation is further compounded by the dominant polarization of Th1/Th17 cells and functional impairments in Treg/Breg cells. Immunotherapy for AA has evolved from a period of broad immunosuppression to a new era focused on targeted immune remodeling. The JAK-STAT signaling pathway, which integrates various cytokine signals, has emerged as a key therapeutic target due to its central regulatory role. Although JAK inhibitors have shown significant clinical effectiveness, therapeutic strategies aiming at specific pathways, such as IL-17 and TNF-α, remain debatable. Future studies should delve deeper into the dynamic interactions among immune cell subsets, investigate combined targeted therapies, and identify more precise biomarkers to tailor personalized treatments. The ultimate aspiration is to attain curative immune tolerance, surpassing mere symptomatic relief.

## Abbreviations

AA: alopecia areata
AASIS: Alopecia Areata Symptom Impact Scale
APC: antigen-presenting cell
4-1BBL: 4-1BB ligand
BMX: bone marrow tyrosine kinase on chromosome X
Bregs: regulatory B cells
BTK: Bruton&rsquo;s tyrosine kinase
γc: gamma chain
cAMP: cyclic adenosine monophosphate
CCL: C-C motif chemokine ligand
CCR: C-C motif chemokine receptor
CD200R: CD200 receptor
CD30L: CD30 ligand
CGRP: calcitonin gene related peptide
CLA: cutaneous lymphocyte-associated antigen
CXCL: C-X-C motif chemokine ligand
CXCR: C-X-C motif chemokine receptor
DAG: diacylglycerol
DCs: dendritic cells
FasL: Fas ligand
FDA: Food and Drug Administration
FoxP3: forkhead box protein 3
α-GalCer: alpha-Galactosylceramide
GZMB: granzyme B
HF-IP: hair follicle immune privilege
HLA-DR: human leukocyte antigen-DR
ICAM1: intercellular adhesion molecule 1
IDO: indoleamine 2,3-dioxygenase
IFN: interferon
IGF-1: insulin-like growth factor-1
IL: interleukin
ILC1: type 1 innate lymphoid cells
IL-1R1: interleukin-1 receptor type 1
IL-15R: interleukin-15 receptor
IL-17RA: interleukin-17 receptor A
iNKT: invariant natural killer T
IP3: inositol trisphosphate
ITK: IL-2-inducible T-cell kinase
JAK: janus kinase
KIRs: killer immunoglobulin-like receptors
MAPK: mitogen-activated protein kinase
MHC: major histocompatibility complex
MICA: major histocompatibility complex class I polypeptide-related sequence A
MIF: macrophage migration inhibitory factor
α-MSH: α-melanocyte-stimulating hormone
NF-κB: nuclear factor-kappa B
NK: natural killer
NKG2D: natural killer cell group 2D
NKT: natural killer T
NLRP3: NOD-like receptor family pyrin domain containing 3
OX40L: OX40 ligand
PBMCs: peripheral blood mononuclear cells
pDCs: Plasmacytoid dendritic cells
PDE4: phosphodiesterase 4
PD-1: programmed cell death-1
PDL1: programmed death ligand 1
PINK1: PTEN induced kinase 1
PIP2: phosphatidylinositol 4,5-bisphosphate
PLCγ1: phospholipase Cγ1
RCTs: randomized controlled trials
RIPK1: receptor-interacting protein kinase 1
RLK: resting lymphocyte kinase, also known as TXK
S1P: sphingosine-1-phosphate
SALT: Severity of Alopecia Tool
SDLNs: skin draining lymph nodes
SIRT1: Sirtuin 1
STAT: signal transducer and activator of transcription
Tc1: type 1 cytotoxic T cell
TCR: T cell receptor
TGF: transforming growth factor
Th: T helper
TLR7/9: Toll-like receptors 7/9
TNF: tumor necrosis factor
Tregs: regulatory T cells
TSLP: thymic stromal lymphopoietin
TSP1: thrombospondin 1
TVM: virtual memory T
TWEAK: tumor necrosis factor-related weak inducer of apoptosis
TYK2: tyrosine kinase 2
ULBP: UL16-binding protein
Wnt/β-catenin: wingless-integrated/beta-catenin
